# Luteolin attenuates doxorubicin-induced cardiotoxicity by modulating the PHLPP1/AKT/Bcl-2 signalling pathway

**DOI:** 10.7717/peerj.8845

**Published:** 2020-05-11

**Authors:** YanDong Zhang, ChengYuan Ma, ChunShui Liu, Feng Wei

**Affiliations:** 1 Department of Rheumatology, First Hospital, Jilin University, ChangChun, Jilin, China; 2 Department of Neurosurgery, First Hospital, Jilin University, ChangChun, Jilin, China; 3 Department of Hematology , First Hospital, Jilin University, ChangChun, Jilin, China; 4 Department of Hepatobiliary & Pancreas Surgery, First Hospital, Jilin University, Changchun, Jilin, China

**Keywords:** Luteolin, Doxorubicin, PH domain leucine-rich repeats protein phosphatase 1 (phlpp1), AKT/Bcl-2 pathway, Cardiotoxicity

## Abstract

**Background:**

Luteolin (LUT) is a flavonoid found in vegetables and fruits that has diverse functions. Doxorubicin (DOX) is an anthracycline antibiotic that is frequently used for the treatment of various cancers. Unfortunately, the clinical efficacy of DOX is limited by its dose-related cardiotoxicity. In this study, we aimed to investigate the potential mechanism through which LUT attenuates cardiotoxicity in vivo.

**Methods:**

We evaluated the body weight, heart weight, electrocardiogram, and pathological changes before and after administration of LUT. Moreover, the effects of LUT (50 mg/kg in the low dose group, 100 mg/kg in the high dose group) on biochemical parameters (brain natriuretic peptide, creatine kinase MB, cardiac troponin T, and dehydrogenation of lactate enzyme) and oxidative stress parameters (malondialdehyde and superoxide dismutase) were studied in the sera of cardiotoxicity model rats. We also identified the apoptotic mediators whose expression was induced by LUT by quantitative real-time reverse transcription-polymerase chain reaction (RT-qPCR) evaluation. In addition, we used network analysis to predict DOX-induced cardiotoxicity and protection afforded by LUT. Western blotting was used to detect the expression of associated proteins.

**Results:**

LUT significantly improved DOX-induced cardiotoxicity in a dose-dependent fashion. LUT ameliorated DOX-induced weight loss and heart weight changes, as well as changes in biochemical parameters and oxidative stress parameters in heart injury model rats. LUT’s protective effect was observed via regulation of the apoptotic markers Bcl-2, Bax, and caspase-3 mRNA and protein expression levels. Network analysis showed that the AKT/Bcl-2 signalling pathway was activated; specifically, the PH domain leucine-rich repeats protein phosphatase 1 (phlpp1) was involved in the AKT/Bcl-2 signal pathway. LUT inhibited the activity of phlpp1 leading to positive regulation of the AKT/Bcl-2 pathway, which attenuated doxorubicin-induced cardiotoxicity.

**Conclusions:**

These results demonstrate that LUT exerted protective effects against DOX-induced cardiotoxicity in vivo by alleviating oxidative stress, suppressing phlpp1 activity, and activating the AKT/Bcl-2 signalling pathway.

## Introduction

**Figure 1:**
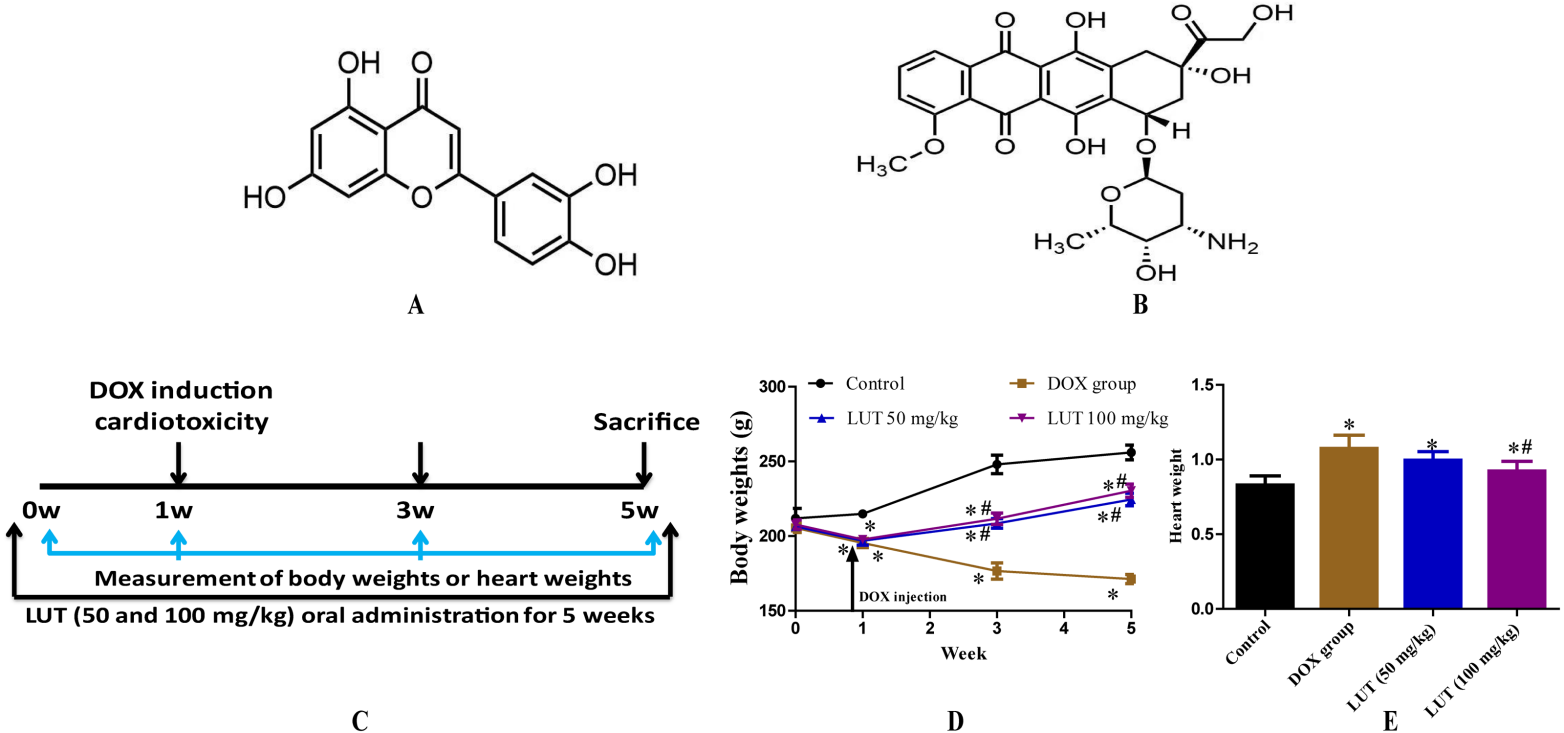
The experimental scheme and the effects of LUT on body weight and heart weight in DOX induced-cardiotoxicity model rats. (A) chemical structure of Luteolin; (B) chemical structure of Doxorubicin; (C) the experimental protocol used to induce cardiotoxicity, followed by the administration of LUT. DOX tail vein injection; (D) body weights; and (E) heart weight. ^∗^ *p* < 0.05 vs. control group; ^#^ *p* < 0.05 vs. the DOX group. LUT, Luteolin; DOX, Doxorubicin.

Doxorubicin (DOX, Fig. 1A) is widely used in the treatment of a variety of malignancies, in particular, solid tumours (Hu et al., 2018). However, it has also been reported to induce toxicity, including myelosuppression (Chen et al., 2017), gastrointestinal reaction (Tomiyasu et al., 2010), and cardiotoxicity (McGowan et al., 2017), thereby, limiting its clinical use (Chung & Youn, 2016; McGowan et al., 2017). The mechanisms underlying anthracycline-induced cardiotoxicity are not fully understood. Studies in recent years have demonstrated that anthracyclines trigger excessive mitochondrial reactive oxygen species (ROS) production in cardiomyocytes, subsequently inducing calcium overload, mitochondrial dysfunction, autophagy dysregulation, and eventually apoptotic and autophagic cell death (Bartlett, Trivedi & Pulinilkunnil, 2017; Cappetta et al., 2016; Ghigo, Li & Hirsch, 2016; Hu et al., 2019; Wang et al., 2019). Dexrazoxane (DZR) is the only drug in clinical use that can protect against DOX-induced cardiotoxicity (Cvetković & Scott, 2005; Zhang et al., 2019). Although DZR effectively reduces the incidence of DOX-induced chronic heart failure (Jones, 2008), it reduces the anti-tumuor effect of anthracyclines (Silber, 2004) and increases the incidence of secondary malignancies (Shaikh et al., 2015). Thus, there is growing interest in the use of new treatments that can delay or treat anthracycline-induced cardiotoxicity. In particular, many studies have indicated that natural products can be potentially used for this purpose (Psotová, 2004; Zhang et al., 2019). The mechanism of cardiotoxicity is multifactorial, and the disease progression is very complicated. A large number of studies have found that doxorubicin causes myocardial damage by blocking the mechanism of antioxidant cells, causing the accumulation of reactive oxygen species and increasing the apoptosis of myocardial cells (Wallace, 2003). Therefore, flavonoids and polyphenols, which are strong antioxidants, have great potential as therapies to alleviate DOX-induced cardiotoxicity.

Luteolin (LUT, Fig. 1B) is an abundant flavonoid found in vegetables and fruits such as celery, broccoli, carrots, and peppers (Pandurangan & Esa, 2014). Luteolin has various biological functions such as anti-inflammatory (Nabavi et al., 2015), antiatherogenic (Kim et al., 2012), and antitumour (Huang, Jin & Lan, 2019) effects. Domitrović et al. (2013) also suggested LUT as an effective nephroprotective agent; specifically, they reported its potential to reduce Pt accumulation in the kidneys and ameliorate cisplatin-induced nephrotoxicity. It has been suggested that cardiotoxicity caused by DOX may be due to the occurrence of oxidative stress. Because LUT is a flavonoid, it has a strong antioxidant effect. Additionally, a large number of studies have found that LUT exerts a protective effect in other heart injury models. Li et al. (2019) suggest that LUT protects heart tissues in STZ-induced diabetic mice by modulating Nrf2-mediated oxidative stress and NF-*κ*B-mediated inflammatory responses. In another study, Yan et al. (2019) demonstrated LUT’s protective effects during long-term heart preservation in a dose-dependent manner, which may be accomplished by inhibiting hypoxia-dependent L-type calcium channels. Yao et al. (2016) also showed Luteolin-7-O-Glucoside cardioprotective effects by inhibiting the DOX-induced intracellular level of ROS and apoptosis, potentially acting as a therapeutic agent for preventing DOX-induced cardiotoxicity. However, because LUT and Luteolin-7-O-Glucoside are two different compounds, it is still unclear whether LUT has pharmacological activity against DOX-induced cardiotoxicity.

Although various biological functions of LUT have been studied, pharmacological analysis of DOX-induced cardiotoxicity with LUT has not been performed. Therefore, we investigated the cardiotoxicity inhibitory effects of LUT in a rat model of DOX-induced cardiotoxicity We then used pharmacological network analysis to assess the overall regulatory mechanism of LUT. Moreover, we identified potential target genes associated with cardiotoxicity to determine the mechanism by which LUT exerted cardioprotective effects.

## Materials & Methods

### Animals

Male Wistar (seven-weeks-old) rats were purchased from the Animal Experimental Center of Jilin University (Changchun, China) and maintained under controlled conditions on 12 h light/dark cycle, 22 °C ± 2 °C, and 55% ±  15% humidity. This study was performed in the School of Pharmacy at Jilin University according to the Guide for the Care and Use of Laboratory Animals. It was approved by the Animal Care and Use Committee of Jilin University [20170503].

### Induction of cardiotoxicity and drug treatment

Cardiotoxicity was induced using doxorubicin (DOX, Shenzhen Main Luck Pharmaceuticals Inc., Shenzhen, China) as previously described (Argun et al., 2016; Mohan et al., 2006). We used sterile water for injection to dissolve DOX and CMC-Na to dissolve LUT for subsequent experiments. Briefly, all groups of rats except the saline (control) group were injected directly into the tail vein with DOX (4 mg/kg, weekly, cumulative dose: 16 mg/kg (Mohan et al., 2006)). This dose was based on the large number of studies that have found that the cumulative dose of 16–20 mg/kg DOX in rats can induce cardiac toxicity. In the previous preliminary experiments, we found that when the cumulative dose reached 20 mg/kg, the mortality rate was increased. Therefore, 16 mg/kg was selected for this experiment. It was also observed that DOX injections twice a week also increased mortality. Based on the above preliminary experiments, we chose the modelling method for this experiment. In this experiment, the dosage of LUT was mainly selected based on previous experiments. Li et al. (2015) demonstrated that luteolin (50 mg/kg and 100 mg/kg) is capable of improving diabetes-induced deficits in motor and sensory functions, which could be attributed in part to its Nrf2-dependent antioxidant capacity. Zhen et al. (2016) found pre-treatment with LUT (50 mg/kg and 100 mg/kg) suppressed seizure induction, duration, and severity following PTZ injection. After one week of adaptive rearing, rats were randomised into four groups (*n* = 8 per group): (1) control group; (2) DOX group; (3) low-dose LUT group (DOX + LUT) with LUT (50 mg/kg) plus DOX injection; and (4) high-dose LUT group (DOX + LUT) with LUT (100 mg/kg) plus DOX injection. The experimental scheme is shown in Fig. 1C. The LUT groups were given the corresponding dose of LUT one week in advance, and the gastric administration lasted for five weeks. The remaining groups were intragastrically administered with a corresponding volume of CMC-Na daily. The injection time of DOX started from 1 w to 4 w, and lasted for four weeks, once a week. The control group was injected with the same volume of saline once a week.

### Measurement of body weight and heart weight

The body weight of the rats was measured at 0, 1, 3, and 5 weeks after DOX induction. In this experiment, DOX injection was performed every Monday, and the body weight of each group of rats was measured every Friday. After five weeks of treatment, the rats were euthanised and blood samples were collected. The heart tissue was immediately removed and weighed to calculate the heart. Half of the heart was used for histological analysis after fixing in 10% buffered formalin, whereas the remaining tissues were frozen in liquid nitrogen and stored at −80 °C until further use.

### Histopathological analysis

The heart tissue was embedded in paraffin and serially sliced. Haematoxylin and eosin (H&E) staining was performed to observe the cells and stroma. Histological changes were examined by light microscopy (Olympus, Olympus Optical Co., Tokyo, Japan) and photographed.

### Electrocardiography

The IV lead electrocardiogram (ECG) was recorded in rats anesthetised by 7% chloral hydrate using the BL-420E Biological Function Measurement System (Chengdu Taimeng Science and Technology Co. Ltd.). Electrodes were inserted into the right upper limb, the lower right limb, and the lower left limb under the skin. Visual analysis of the recorded ECG was performed by two experts to assess the heart rate and ECG abnormalities.

### Measurement of biochemical indices

Determination of brain natriuretic peptide (BNP), creatine kinase MB (CK-MB), cardiac troponin T (CTnT), malondialdehyde (MDA), dehydrogenation of lactate enzyme (LDH), and superoxide dismutase (SOD) were evaluated using ELISA kits (Nanjing JianCheng Biological Engineering Research Institute, Nanjing China) according to the manufacturer’s instructions.

### Identification of DOX-induced cardiotoxicity associated proteins and LUT-associated target genes

Information on DOX-induced cardiotoxicity-associated gene targets and LUT related genes were identified from the Comparative Toxicogenomics Database (CTD, http://ctdbase.org/), which is a robust, publicly available database that provides comprehensive, user-friendly information on chemical-gene/protein interactions, and chemical-disease and gene-disease relationships.

### Network construction and analysis

To investigate the interaction between LUT and DOX-induced cardiotoxicity target genes, a network was constructed using the network visualization software Cytoscape ver. 3.5.1 (Smoot et al., 2011). The software is used to visualize biological pathways and molecular interaction networks as well as for data integration, analysis, and visualization/analysis of complex networks. In the network, nodes represent compounds or target genes, and edges represent compound-target gene interactions. After the network analysis, the database (DAVID), ver 6.8 was used for annotation, visualization, and integration discovery. Functional annotation of the gene was performed using DAVID 6.8 and the Kyoto Encyclopedia of Genes and Genomes (KEGG).

### Real-time quantitative RT-PCR analysis

Total RNA was extracted from the cardiac tissue using TRIzol reagent (Takara, Dalian, China), reverse transcribed into cDNA, and amplified by PCR using TransScript Green two-step qRT-PCR Supermix (TransGen Biotech, Beijing, China). Real-time quantitative PCR was performed using a real-time PCR system. Aliquots of sample cDNA and equal amounts of β-actin cDNA were amplified using a master mix containing DNA polymerase, according to the manufacturer’s instructions. The PCR amplification cycle conditions were 50 °C for 2 min, 94 °C for 10 min, 95 °C for 15 s, and 60 °C for 1 min for 40 cycles. The relative expression of the target gene was determined using the method of comparing Ct (the number of threshold cycles at the intersection between the amplification curve and the threshold), according to the manufacturer’s instructions. The sequences of the primers and probes used are listed in Table 1.

**Table 1:** Sequences of real-time PCR primers.

**Gene**	**Primer Sequence (5**′–3′)	**Product size (bp)**	**Accession Number**
Bcl-2	F: GGGATGCCTTTGTGGAACTA R: CTCACTTGTGGCCCAGGTAT	138	NM_016993.1
Bax	F: TGTTTGCTGATGGCAACTTC R: GATCAGCTCGGGCACTTTAG	104	NM_017059.1
Caspase-3	F:GGTATTGAGACAGACAGTGG R:CATGGGATCTGTTTCTTTGC	393	NM_012922.2
β-actin	F: GCCATGTACGTAGCCATCCA R: GAACCGCTCATTGCCGATAG	374	NM_031632

### Western blot analysis

Heart tissue protein levels were analysed by western blotting. Anti-phlpp1, anti-AKT, anti-p-AKT, anti-cleaved-caspase-3, anti-Bcl-2, anti-Caspase-3, anti-GAPDH, anti-Bax and secondary antibodies were purchased from Abcam (MA, USA). Briefly, heart tissue was disrupted and then rapidly homogenised in 200 µL RIPA lysis buffer. Protein concentrations were determined by the BCA method. An equal amount of protein sample was separated by sodium dodecyl sulphate-polyacrylamide gel electrophoresis (SDS-PAGE) and transferred to a nitrocellulose membrane. The membrane was blocked with 5% milk in Tris buffered saline (TBS) and then incubated with anti-Bax (1:1,000 dilution), anti-Bcl-2 (1:500 dilution), anti-pro-caspase 3 (1:1,000 dilution), anti-cleaved-caspase-3 (1:1,000 dilution), anti-AKT (1:1,000 dilution), anti-p-AKT (1:1,000 dilution), anti-phlpp1 (1:1,000 dilution), and anti-GAPDH (1:1,000 dilution) antibody. The membrane was incubated overnight at 4 °C, washed three times, and then incubated with the respective second antibody (1:5,000) for 60-90 min. Protein bands were visualised using enhanced chemiluminescent reagents. Protein expression levels were analysed using a digital gel imaging system (Alpha Imager 2200, Alpha Innotech Corporation, San Leandro, CA, USA).

### Statistical analysis

All results are expressed as the mean ± standard error of the mean (SEM). Statistical analysis was performed using one-way analysis of variance, multiple comparisons were performed using Tukey’s multiple comparison test, and *p* < 0.05 was considered statistically significant. Statistical analysis was performed using GraphPad Prism software version 5.0 for Windows.

## Results

### LUT administration restored the heart and body weight in rats with DOX-induced cardiotoxicity

To evaluate the cardioprotective effects of LUT on cardiotoxicity, we assessed the body weight of rats for five weeks and the heart weight at euthanasia after DOX induction. As shown in Figs. 1D and 1E, with DOX alone, the body weights of rats were significantly decreased (*p* < 0.05) and the heart weights were significantly increased (*p* < 0.05) as compared with those of rats in the control group. This showed that DOX could significantly change the weight of the heart and affect heart function in vivo. However, LUT significantly attenuated these effects (*p* < 0.05). These results demonstrated that LUT alleviated changes in heart weight induced by DOX.

### LUT treatment recovered the histopathological features of heart tissue in DOX-induced cardiotoxicity

**Figure 2:**
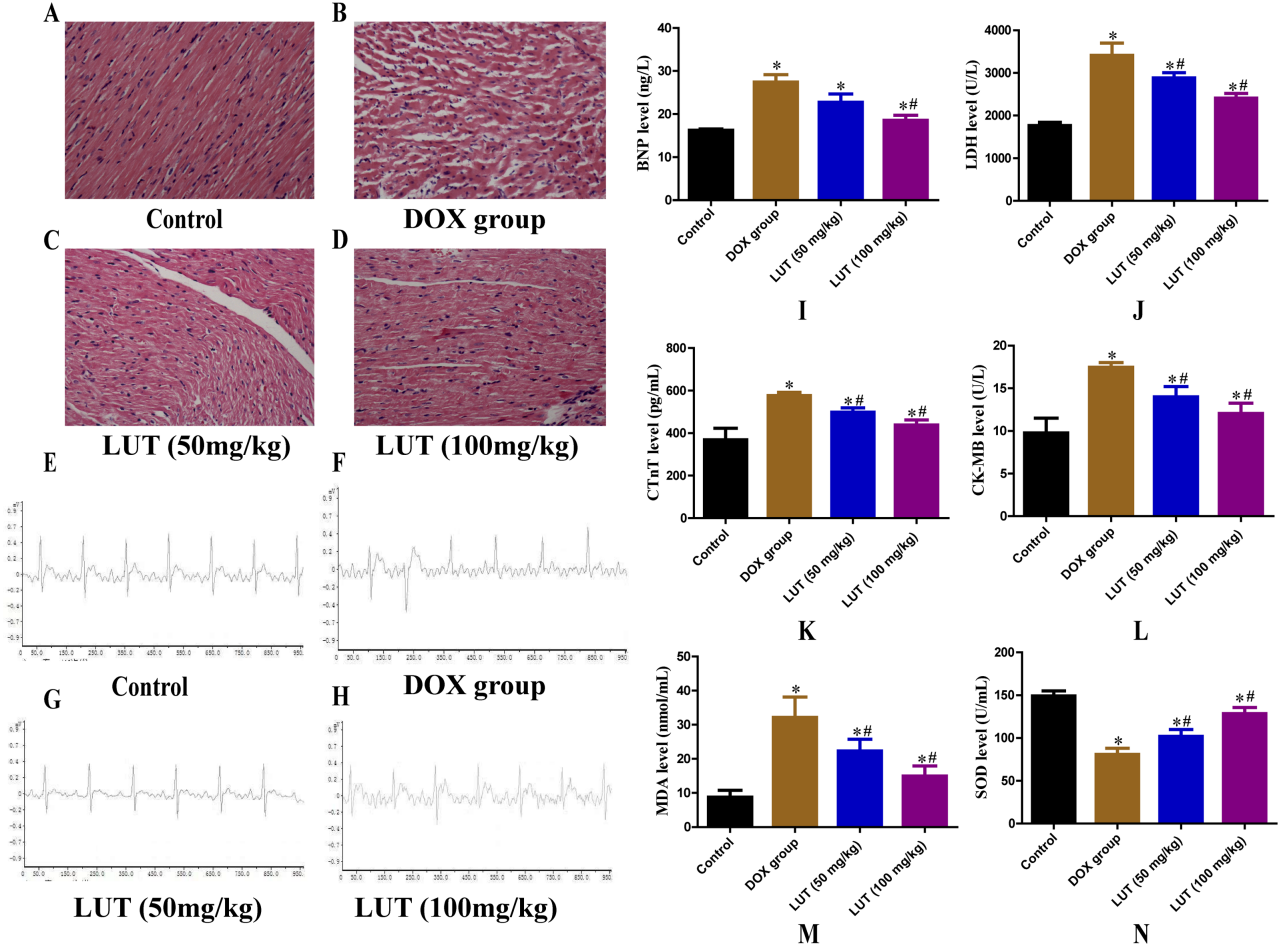
Effects of LUT on the histopathological features, electrocardiogram, serum levels of cardiac injury, and oxidative stress mediators in the heart tissue of DOX-induced cardiotoxicity model rats. (A–D) Haematoxylin and Eosin staining (×200 magnification). (E–H) Electrocardiogram. (I) Representative brain natriuretic peptide levels (BNP). (G) Representative lactate dehydrogenase levels (LDH). (K) Representative cardiac troponin T levels (CTnT). (L) Representative creatine kinase MB levels (CK-MB). (M) Representative malondialdehyde (MDA) levels. (N) Representative superoxide dismutase (SOD) levels. ^∗^ *p* < 0.05 vs. the control group; ^#^ *p* < 0.05 vs. the DOX group. LUT, Luteolin; DOX, Doxorubicin.

We investigated whether LUT could exert a therapeutic effect in vivo using a DOX-induced rat model. Heart sections were stained with H&E (Figs. 2A– 2D). A regular distribution of cardiomyocytes was observed in the control group without any significant histopathological changes. As expected, DOX caused a significant increase in intracellular space, cytoplasmic vacuolation, and myocardial cell disorders. However, pathological changes in the myocardium were significantly attenuated in rats treated with different doses of LUT. In particular, pre-treatment with LUT at a dose of 100 mg/kg attenuated DOX-induced histopathological changes.

### LUT improved electrocardiogram changes

Electrocardiograms of rats from each group were analysed (Figs. 2E– 2H). Rats in the control group showed no significant changes in the heart rate, limb voltage, and QRS interval. Rats in the DOX (alone) group showed a lower heart rate, decreased R wave voltage, and prolonged QT interval. Pre-treatment with LUT at a dose of 50 mg/kg or 100 mg/kg showed a significant improvement in the heart rate, R wave amplitude, and QT interval prolongation. In particular, LUT at a dose of 100 mg/kg significantly attenuated these ECG changes.

### LUT reduced serum marker levels of heart damage upon cardiac injury

Serum markers of heart damage are key enzymes in the heart that are released into the blood when cardiomyocytes are damaged (Wang et al., 2018). Plasma BNP, CK-MB, CTnT, and LDH activities were significantly elevated in the DOX alone group (*p* < 0.05), confirming the cardiotoxicity of DOX (Figs. 2I–2L). However, serum marker levels of cardiac damage in LUT-treated (50 mg/kg and 100 mg/kg) rats were significantly improved as compared to those in the DOX-only treatment group (*p* < 0.05).

### LUT inhibited DOX-induced oxidative stress

Some studies have reported that DOX-induced cardiotoxicity is associated with oxidative stress (Cappetta et al., 2017). We examined the effect of LUT on SOD and MDA levels as the primary parameter for assessing free radical metabolism (Figs. 2M and 2N). After exposure to DOX, SOD levels were significantly reduced (*p* < 0.05) and MDA levels were significantly increased (*p* < 0.05) compared to the control group. However, pre-treatment with LUT significantly inhibited MDA levels and increased SOD levels in myocardial tissue (*p* < 0.05). The most significant increase in SOD levels was observed in the group pre-treated with 100 mg/kg LUT (*p* <0.05), indicating that LUT has dose-dependent cardioprotective and antioxidant functions in vivo.

### LUT Treatment inhibited mRNA expression levels of apoptosis mediators in DOx-induced cardiotoxicity rats

**Figure 3:**
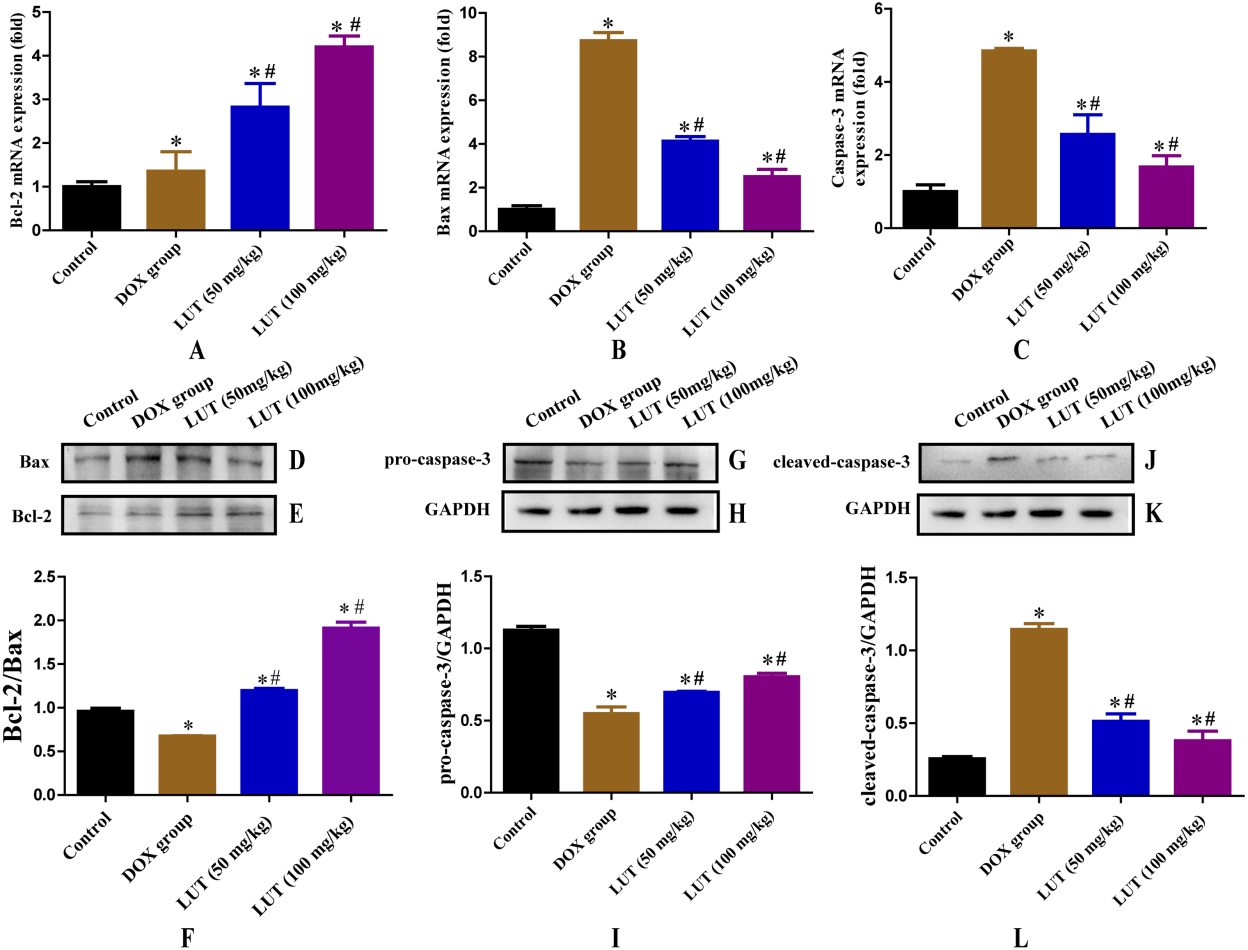
Effects of LUT on the expression of apoptotic factors in the heart tissue of DOX-induced cardiotoxicity model rats. (A) Representative Bcl-2 mRNA expression. (B) Representative Bax mRNA expression. (C) Representative Casp3 mRNA expression. (D–F) Representative Bcl-2 and Bax protein ratio. (G–I) Representative pro-caspase-3 and GAPDH protein ratio. (J–L) Representative Cleaved-caspase-3 and GAPDH protein ratio. ^∗^ *p* < 0.05 vs. the control group; # *p* < 0.05 vs. the DOX group. LUT, Luteolin; DOX, Doxorubicin.

We examined the effect of LUT on the expression of apoptosis mediators (caspase-3, Bcl-2, and Bax) in DOX-induced rat myocardial tissue. As shown in Figs. 3A–3C, after exposure to DOX, the mRNA expression levels of caspase-3 and Bax and Bcl-2 were significantly increased (*p* <  0.05) compared to the control group. However, pre-treatment with LUT the mRNA expression levels of caspase-3 and Bax were significantly decreased (*p* < 0.05), whereas those of Bcl-2 were significantly increased (*p* < 0.05) compared to the DOX group.

### LUT treatment increased the Bcl2/bax ratio

Bcl-2 and Bax play antagonistic roles in apoptosis; Bcl-2 inhibits apoptosis, whereas Bax promotes apoptosis. Therefore, the Bcl-2/Bax ratio is a key factor in regulating apoptosis. Representative Bax and Bcl-2 western blots are shown in Figs. 3D– 3F. Compared with the control group, DOX treatment significantly reduced the Bcl-2/Bax protein ratio (*p* < 0.05). Treatment with LUT, however, significantly increased the Bcl-2/Bax ratio (*p* < 0.05) in a dose-dependent fashion.

### LUT reduced the expression of caspase family proteins

Caspase family proteins induce apoptosis in cardiomyocytes. Representative images of pro-caspase-3 and cleaved-caspase-3 protein levels are shown in Figs. 3G–3L. As expected, DOX treatment significantly increased the level of cleaved-caspase-3 as compared to that in the control group (*p* < 0.05), and decreased the level of pro-caspase-3 (*p* < 0.05). However, treatment with LUT resulted in increased pro-caspase-3 expression (*p* < 0.05), and decreased expression of cleaved-caspase-3 as compared to the DOX group, indicating reduced apoptosis (*p* < 0.05).

### In silico network analysis and prediction of target genes and pathways related to cardiotoxicity

To further elucidate the interaction between LUT and target genes, relationships were investigated using network analysis. The LUT resulting network included 142 potential target genes as shown in Table S1. We then used the network analysis to find the interaction between DOX and target genes. The network for DOX included a total of 364 genes (Inference Score ≥ 4), indicating a higher correlation with cardiotoxicity. A total of 50 genes were associated with LUT and DOX (Table S2).

**Figure 4:**
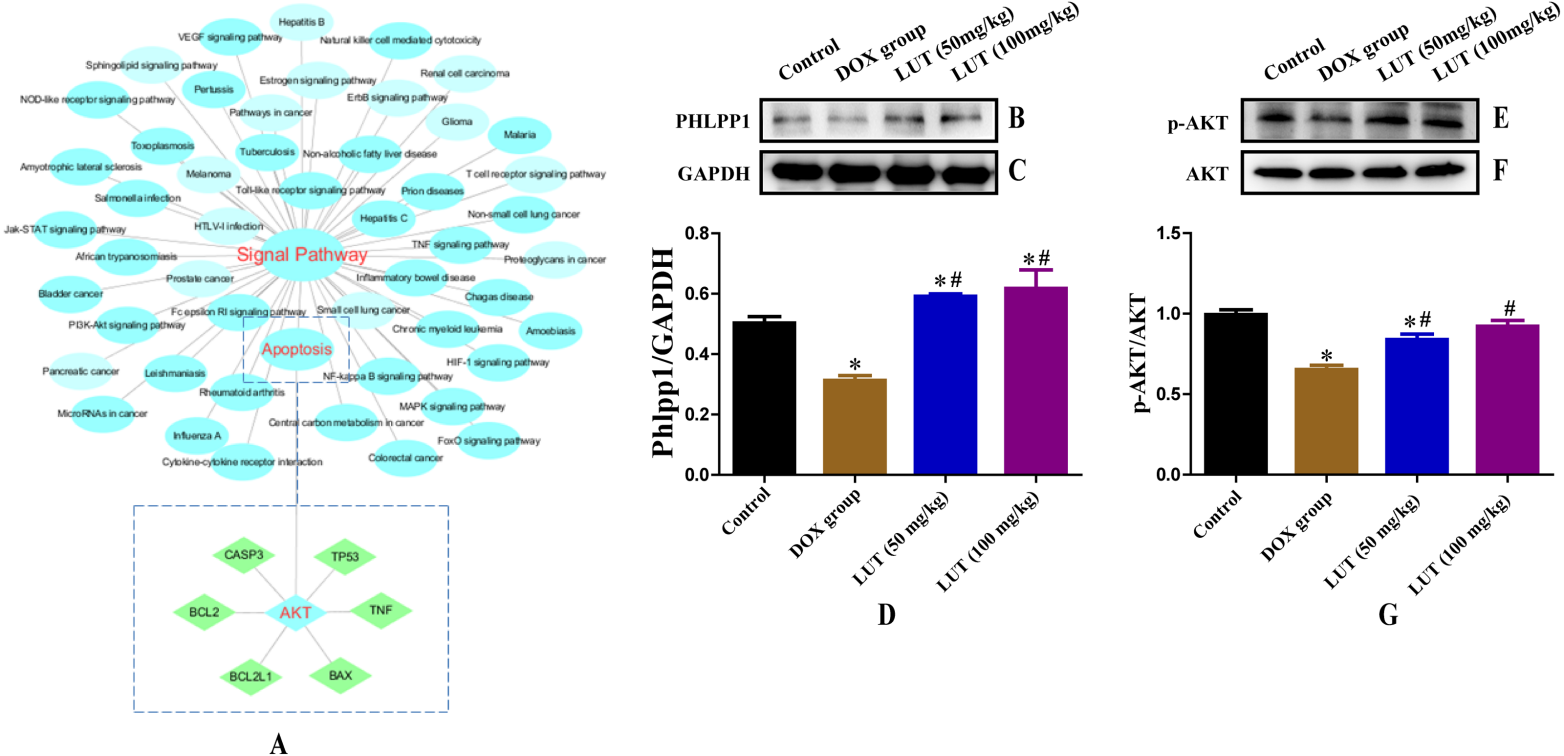
Chemicals-target gene network linking the protective effects of LUT against cardiotoxicity to potential signalling pathways and related protein expression. (A) Blue circles represent the enriched KEGG main pathway, and green rectangles represent the top putative target proteins. (B–D) Representative phlpp1 and GAPDH protein ratio. (E–G) p-AKT and AKT protein ratio. ^∗^ *p* < 0.05 vs. the control group; ^#^ *p* < 0.05 vs. the DOX group. LUT, Luteolin; DOX, Doxorubicine.

To better understand the signaling pathways and functions of these target genes, we performed functional enrichment analysis using DAVID software and the KEGG database. Potential target genes were functionally related to various signal transduction pathways (Fig. 4A; Table 2), particularly those regulated by p-AKT. Therefore, the expression of p-AKT was detected in subsequent experiments.

### LUT increased phlpp1 protein expression

When dephosphorylation occurs in phlpp1, phosphorylation of AKT is inhibited and apoptosis is induced (Specific KEGG diagram: https://www.kegg.jp/kegg-bin/show_pathway?rno04151+498949). Representative images of phlpp1 protein expression are shown in Figs. 4B–4D. DOX treatment significantly decreased the level of phlpp1 as compared to the control group (*p* < 0.05). However, treatment with LUT increased phlpp1 expression as compared to the DOX group (*p* < 0.05). This indicated that LUT could inhibit the phosphorylation of phlpp1, thereby reducing the induction of apoptosis.

**Table 2:** Kyoto Encyclopedia of Genes and Genomes (KEGG) pathways and target genes of LUT potentially responsible for the therapeutic activities against cardiotoxicity.

**Pathway Classification**	**Pathway ID**	**Term**	**Target Gene**
Signal transduction	hsa04066	HIF-1 signaling pathway	AKT1, CASP3, FAS, FGF2, EGFR, MAPK1, MAPK14, VEGFA, MAPK3
Signal transduction	hsa04152	AMPK signaling pathway	AKT1, IGF1R, SLC2A4, PPARG, FASN, IGF1, ADIPOQ, SIRT1
Signal transduction	hsa04150	mTOR signaling pathway	PRKCA, AKT1, MAPK1, TNF, MAPK3, IGF1
Inflammation-generating process	hsa04668	TNF signaling pathway	IL6, TNF, AKT1, MAPK1, CASP3, CASP8, MAPK3, IL1B, FAS
Inflammation-generating process	hsa04370	VEGF signaling pathway	AKT1, MAPK1, PTK2, PTGS2, MAPK14, MAPK3, VEGFA, RAC1, NOS3
Apoptosis	hsa04151	PI3K-Akt signaling pathway	AKT1, BCL2, IL4, IL6, TP53, MAPK1, VEGFA, MAPK3, IL2
Apoptosis	hsa04068	FoxO signaling pathway	IL6, SOD2, AKT1, MAPK1, CDKN1A, SLC2A4, MAPK14, MAPK3, CAT
Apoptosis	hsa04210	Apoptosis	AKT1, CASP3, TNF, BAX, BCL2, CASP8, FAS, ATM
Apoptosis	hsa04115	p53 signaling pathway	CASP3, BAX, CASP8, TP53, IGF1, CDK6, FAS, ATM
Apoptosis	hsa04010	MAPK signaling pathway	TNF, TP53, AKT1, MAPK1, CASP3, MAPK3, FAS, IL1A
Apoptosis	hsa04014	Ras signaling pathway	IGF1, HGF, AKT1, MAPK1, IGF1R, VEGFA, MAPK3, RAC1, FGF1

### LUT increased p-AKT protein expression

Through network analysis, we found that AKT can regulate the expression of apoptotic factors. Therefore, we assessed the expression of AKT and p-AKT. Representative images of AKT and p-AKT protein expression are shown in Figs. 4E–4G. DOX treatment significantly decreased the level of p-AKT as compared to that in the control group (*p* < 0.05), whereas treatment with LUT increased p-AKT expression as compared to that in the DOX only group (*p* < 0.05).

## Discussion

Doxorubicin is frequently used in chemotherapy against acute leukaemia, malignant lymphoma, and several solid tumours. Although DOX has been frequently reported to exhibit minimal cardiotoxicity at a single dose, there is still a risk of cardiomyopathy when multiple doses are administered (Hallman et al., 2019). The pathophysiological mechanisms underlying doxorubicin-induced cardiotoxicity include three interrelated respects: accumulation of reactive oxygen species (ROS); dysfunction of topoisomerase II- β and topoisomerase I; and mitochondrial imbalance of intracellular calcium (Burridge et al., 2016). Among these, ROS play a critical role in that they can cause functional and structural damage to the cell (Dai et al., 2018). Oxidative stress is considered the major factor involved in DOX-induced cardiotoxicity. The organelle most significantly affected is the mitochondria due to DOX accumulation at the inner membrane combining with cardiolipin, an essential ingredient for the electron-transport chain, thereby fuelling the production of ROS. Although it is generally accepted that ROS are the main cause of cell apoptosis, it is known that apoptosis can occur independently of ROS after administration of DOX (Octavia et al., 2012). Considering its mechanism, administration of antioxidant supplements may be key components in cardio-oncology signaling (Lipshultz et al., 2013). To investigate the efficacy of flavonoids for drug-induced cardiotoxicity, we attempted to validate the efficacy of LUT in a DOX-induced cardiotoxicity rat model. Moreover, we explored the potential molecular mechanisms of the LUT by using a systematic network analysis approach and verified the predicted results by experimental pharmacological analysis.

Myocardial histopathological alterations were attenuated to a notable extent in rats treated with different doses of LUT. Pre-treatment with LUT at a dose of 50 mg/kg or 100 mg/kg showed marked improvements in heart rate, amplitude of R-wave, and prolonged QT intervals. Cardiac markers such as CTnT, LDH, CK-MB, and brain BNP have been used clinically as sensitive diagnostic markers of myocardial necrosis. The elevated levels of cardiac markers in the DOX group suggested that myocardial injury was induced by DOX. However, when DOX was administered after LUT pre-administration, the serum marker levels were reduced. In line with our findings, LUT has also been reported to play a cardioprotective role in other disease models. For example, Zhang et al. (2017). found that LUT prevented SI/R-induced myocardial damage by reducing oxidative stress-induced injury in isolated rat hearts and cardiomyocytes.

In the present study, MDA levels were elevated, whereas SOD levels were decreased after treatment with DOX, which indicated oxidative stress (Zhang et al., 2018). However, LUT significantly reduced MDA and increased SOD levels, indicating that LUT has antioxidant properties. A similar effect has been reported in a previous study in which LUT could significantly decrease renal MDA and 8- OHdG levels, but significantly improved the SOD and CAT activities in the I/R group (Hong et al., 2017).

Through our network analysis, we identified that key target genes of LUT were AKT, CASP3, Bax, and Bcl, which play a key role in cardiotoxicity. These major nodes identified by the KEGG pathway analysis are associated with apoptotic signalling and are known to be associated with cardiotoxicity. AKT, a well-known pro-survival kinase, is activated by phosphorylation at Ser 473. This activation is known to play a critical role in cell survival (Li et al., 2013). When AKT is phosphorylated, it can promote cell survival by enhancing the function of the antiapoptotic molecule Bcl-2 (Takada-Takatori et al., 2006). Bcl-2 inhibits apoptosis by preventing the release of cytochrome c and the subsequent activation of caspases (Cory, Huang & Adams, 2003). Previous studies have shown that ketamine induces neuronal apoptosis through down-regulation of Bcl-2 expression (Huang et al., 2012). Liu et al. (2011) reported that 17b-estradiol exerts neuroprotective effects against oxidative toxicity by up-regulating Bcl-2 expression. Recently, phlpp, a novel family of Ser/Thr protein phosphatases, has been reported to be able to negatively regulate AKT (Warfel & Newton, 2012). Phlpp members directly dephosphorylate AKT and terminate the downstream signalling pathway. Chen et al. (2013) found that deletion of phlpp1 can enhance AKT activation in neurons and astrocytes, and can significantly increase cell survival and diminish infarct size after MCAO. Based on these findings, inhibition of phlpp could be a therapeutic approach to minimize damage after focal ischaemia.

In this study, we also analysed the mRNA levels of the apoptotic markers Bax, Bcl-2, and caspase-3. Treatment with DOX significantly increased Bax and caspase-3 mRNA, and decreased Bcl-2 mRNA expression as compared to the control group. However, treatment with LUT significantly reduced Bax and caspase-3 mRNA expression as compared to the DOX alone group. These results indicated that LUT could reduce the expression of apoptotic factors in the cytoplasm and prevent apoptosis.

We also observed changes in p-AKT, phlpp1, Bax, Bcl-2, and caspase-3 protein expression levels after treatment with DOX and LUT. Treatment with DOX significantly increased cleaved-caspase-3 protein expression and decreased p-AKT, phlpp1, and Bcl-2/Bax ratio as compared to the control group. Treatment with LUT significantly increased the p-AKT, phlpp1, Bcl-2/Bax ratio, and pro-caspase-3, but decreased the cleaved caspase-3 levels as compared to the DOX only group. These results indicated that LUT could inhibit the activity of phlpp1, leading to positive regulation of the AKT/Bcl-2 pathway, attenuating doxorubicin-induced cardiotoxicity. Collectively, the combined use of experimental animal models and network analysis confirmed that LUT has beneficial therapeutic effects on DOX-induced cardiotoxicity. Moreover, it identified intracellular signalling pathways and target genes associated with therapeutic effects of LUT.

## Conclusions

Overall, we find that LUT exerted its protective effect against doxorubicin-induced cardiotoxicity injuries not only through inhibition of the ROS-mediated oxidative stress but also through inhibition of phlpp1 activity, leading to positive regulation of the AKT/Bcl-2 pathway. Therefore, these findings suggest oral LUT administration may be a novel, natural therapeutic to protect against chemotherapy-induced cardiotoxicity.

##  Supplemental Information

10.7717/peerj.8845/supp-1 Supplemental Information 1 Supplemental Tables **Table S1.** List of 142 potential LUT-related target genes
**Table S2.** List of 50 both LUT and DOX have genes

10.7717/peerj.8845/supp-2 Supplemental Information 2 Body weight and heart weight

10.7717/peerj.8845/supp-3 Supplemental Information 3 Rat serum related factor data

10.7717/peerj.8845/supp-4 Supplemental Information 4 Pcr and western blot related data

10.7717/peerj.8845/supp-5 Supplemental Information 5 Bax protein band

10.7717/peerj.8845/supp-6 Supplemental Information 6 Bcl-2 protein band

10.7717/peerj.8845/supp-7 Supplemental Information 7 Pro-caspase-3 protein band

10.7717/peerj.8845/supp-8 Supplemental Information 8 Cleaved-caspase-3 protein band

10.7717/peerj.8845/supp-9 Supplemental Information 9 Pro-caspase-3 and cleaved-caspase-3 Internal reference strip

10.7717/peerj.8845/supp-10 Supplemental Information 10 AKT protein expression band

10.7717/peerj.8845/supp-11 Supplemental Information 11 AKT protein expression band

10.7717/peerj.8845/supp-12 Supplemental Information 12 phlpp1 protein expression band

10.7717/peerj.8845/supp-13 Supplemental Information 13 Phlpp1 internal reference strip Only the first four bands were used in the experiment.

10.7717/peerj.8845/supp-14 Supplemental Information 14 Bcl2 and Bax data

## Additional Information and Declarations

### Data Availability

The following information was supplied regarding data availability: The raw data are available in the Supplementary Files.
