# Supplementary material for: Luteolin attenuates doxorubicin-induced cardiotoxicity by modulating the PHLPP1/AKT/Bcl-2 signalling pathway
Source: PeerJ. 2020 May 11;8:e8845. doi: 10.7717/peerj.8845 (PMC7224230; doi:10.7717/peerj.8845)
Supplement: Supplemental Information 1 — Table S1. List of 142 potential LUT-related target genes Table S2. List of 50 both LUT and DOX have genes [file peerj-08-8845-s001.doc]

**Supplementary Material**

**Table S1.** List of 142 potential LUT-related target genes

| **Gene Symbol** | **Gene ID** | **Interaction Count** |
| --- | --- | --- |
| TNF | 7124 | 38 |
| IL1B | 3553 | 22 |
| AKT1 | 207 | 14 |
| CYP1A1 | 1543 | 14 |
| MAPK1 | 5594 | 13 |
| MAPK3 | 5595 | 12 |
| CASP3 | 836 | 11 |
| IL6 | 3569 | 11 |
| PTGS2 | 5743 | 11 |
| EGF | 1950 | 8 |
| EGFR | 1956 | 8 |
| IFNG | 3458 | 8 |
| MMP9 | 4318 | 8 |
| RELA | 5970 | 8 |
| NFKBIA | 4792 | 7 |
| HIF1A | 3091 | 6 |
| INS1 | 16333 | 6 |
| JUN | 3725 | 6 |
| NOS2 | 4843 | 6 |
| VEGFA | 7422 | 6 |
| BCL2 | 596 | 5 |
| CASP9 | 842 | 5 |
| CCNB1 | 891 | 5 |
| CDKN1A | 1026 | 5 |
| IRF3 | 3661 | 5 |
| MAOA | 4128 | 5 |
| BAX | 581 | 4 |
| CASP8 | 841 | 4 |
| CYP1B1 | 1545 | 4 |
| FOS | 2353 | 4 |
| HMOX1 | 3162 | 4 |
| ICAM1 | 3383 | 4 |
| IL4 | 3565 | 4 |
| MAPK8 | 5599 | 4 |
| PTK2 | 5747 | 4 |
| STAT3 | 6774 | 4 |
| TP53I3 | 9540 | 4 |
| AHR | 196 | 3 |
| CD40 | 958 | 3 |
| ESR1 | 2099 | 3 |
| GCLC | 2729 | 3 |
| NFE2L2 | 4780 | 3 |
| TNFSF10 | 8743 | 3 |
| ABCC1 | 4363 | 2 |
| ABCC2 | 1244 | 2 |
| ACTA2 | 59 | 2 |
| BCL2L1 | 598 | 2 |
| CAT | 847 | 2 |
| CCL2 | 6347 | 2 |
| CDH1 | 999 | 2 |
| CDH2 | 1000 | 2 |
| CDK2 | 1017 | 2 |
| CSF2 | 1437 | 2 |
| CXCL10 | 3627 | 2 |
| CXCL8 | 3576 | 2 |
| CXCL9 | 4283 | 2 |
| CYP3A4 | 1576 | 2 |
| DHRS11 | 79154 | 2 |
| ESR2 | 2100 | 2 |
| GADD45B | 4616 | 2 |
| GCLM | 2730 | 2 |
| IFNB1 | 3456 | 2 |
| IL10 | 3586 | 2 |
| IL12B | 3593 | 2 |
| IL27 | 246778 | 2 |
| MAPK9 | 5601 | 2 |
| MMP1 | 4312 | 2 |
| NFKB1 | 4790 | 2 |
| PARP1 | 142 | 2 |
| PSCA | 8000 | 2 |
| SELE | 6401 | 2 |
| SFN | 2810 | 2 |
| SLC16A1 | 6566 | 2 |
| SLC5A1 | 6523 | 2 |
| SOD1 | 6647 | 2 |
| TBK1 | 29110 | 2 |
| TP63 | 8626 | 2 |
| TP73 | 7161 | 2 |
| TYR | 7299 | 2 |
| UGT1A1 | 54658 | 2 |
| UGT1A3 | 54659 | 2 |
| VCAM1 | 7412 | 2 |
| XDH | 7498 | 2 |
| ABCC4 | 10257 | 1 |
| ABCG2 | 9429 | 1 |
| ACE | 1636 | 1 |
| AGER | 177 | 1 |
| ALOX5 | 240 | 1 |
| BIRC2 | 329 | 1 |
| CASP1 | 834 | 1 |
| CASP7 | 840 | 1 |
| CCN5 | 8839 | 1 |
| CCNA2 | 890 | 1 |
| CD40LG | 959 | 1 |
| CD74 | 972 | 1 |
| CDK1 | 983 | 1 |
| CDKN1B | 1027 | 1 |
| CDKN2C | 1031 | 1 |
| CHUK | 1147 | 1 |
| CYP1A2 | 1544 | 1 |
| CYP2C19 | 1557 | 1 |
| CYP2C9 | 1559 | 1 |
| CYP2E1 | 1571 | 1 |
| CYP3A5 | 1577 | 1 |
| EGR1 | 1958 | 1 |
| FBP1 | 2203 | 1 |
| FGF2 | 2247 | 1 |
| FOSB | 2354 | 1 |
| GFAP | 2670 | 1 |
| GPR35 | 2859 | 1 |
| GPX1 | 2876 | 1 |
| HBEGF | 1839 | 1 |
| IL13 | 3596 | 1 |
| IL17A | 3605 | 1 |
| IL3 | 3562 | 1 |
| IL5 | 3567 | 1 |
| INSR | 3643 | 1 |
| JUNB | 3726 | 1 |
| JUND | 3727 | 1 |
| MAPK10 | 5602 | 1 |
| MDM2 | 4193 | 1 |
| MMP13 | 4322 | 1 |
| MPO | 4353 | 1 |
| NFAT5 | 10725 | 1 |
| NFKBIB | 4793 | 1 |
| NLRP1A | 195046 | 1 |
| NOS3 | 4846 | 1 |
| NR1D1 | 9572 | 1 |
| P2RX4 | 5025 | 1 |
| PKM | 5315 | 1 |
| PLK1 | 5347 | 1 |
| PRKCA | 5578 | 1 |
| PYCARD | 29108 | 1 |
| SLC2A3 | 6515 | 1 |
| SLC2A4 | 6517 | 1 |
| TGFB1 | 7040 | 1 |
| TNFAIP3 | 7128 | 1 |
| TOP1 | 7150 | 1 |
| TP53 | 7157 | 1 |
| TRP53 | 22059 | 1 |
| VAV3 | 10451 | 1 |
| XIAP | 331 | 1 |

**Table S2.** List of 50 both LUT and DOX have genes

| **Gene Symbol** | **Gene ID** |
| --- | --- |
| TNF | 7124 |
| IL1B | 3553 |
| AKT1 | 207 |
| CYP1A1 | 1543 |
| MAPK1 | 5594 |
| MAPK3 | 5595 |
| CASP3 | 836 |
| IL6 | 3569 |
| PTGS2 | 5743 |
| EGFR | 1956 |
| IFNG | 3458 |
| MMP9 | 4318 |
| HIF1A | 3091 |
| NOS2 | 4843 |
| VEGFA | 7422 |
| BCL2 | 596 |
| CDKN1A | 1026 |
| MAOA | 4128 |
| BAX | 581 |
| CYP1B1 | 1545 |
| HMOX1 | 3162 |
| ICAM1 | 3383 |
| IL4 | 3565 |
| PTK2 | 5747 |
| STAT3 | 6774 |
| ESR1 | 2099 |
| GCLC | 2729 |
| NFE2L2 | 4780 |
| BCL2L1 | 598 |
| CAT | 847 |
| CCL2 | 6347 |
| CSF2 | 1437 |
| CXCL8 | 3576 |
| ESR2 | 2100 |
| GCLM | 2730 |
| IL10 | 3586 |
| PARP1 | 142 |
| SELE | 6401 |
| SOD1 | 6647 |
| XDH | 7498 |
| ACE | 1636 |
| CYP2E1 | 1571 |
| FGF2 | 2247 |
| GPX1 | 2876 |
| NOS3 | 4846 |
| PRKCA | 5578 |
| SLC2A4 | 6517 |
| TGFB1 | 7040 |
| TP53 | 7157 |
| TRP53 | 22059 |
