# Supplementary figures and images for: Luteolin attenuates doxorubicin-induced cardiotoxicity by modulating the PHLPP1/AKT/Bcl-2 signalling pathway
Source: PeerJ. 2020 May 11;8:e8845. doi: 10.7717/peerj.8845 (PMC7224230; doi:10.7717/peerj.8845)

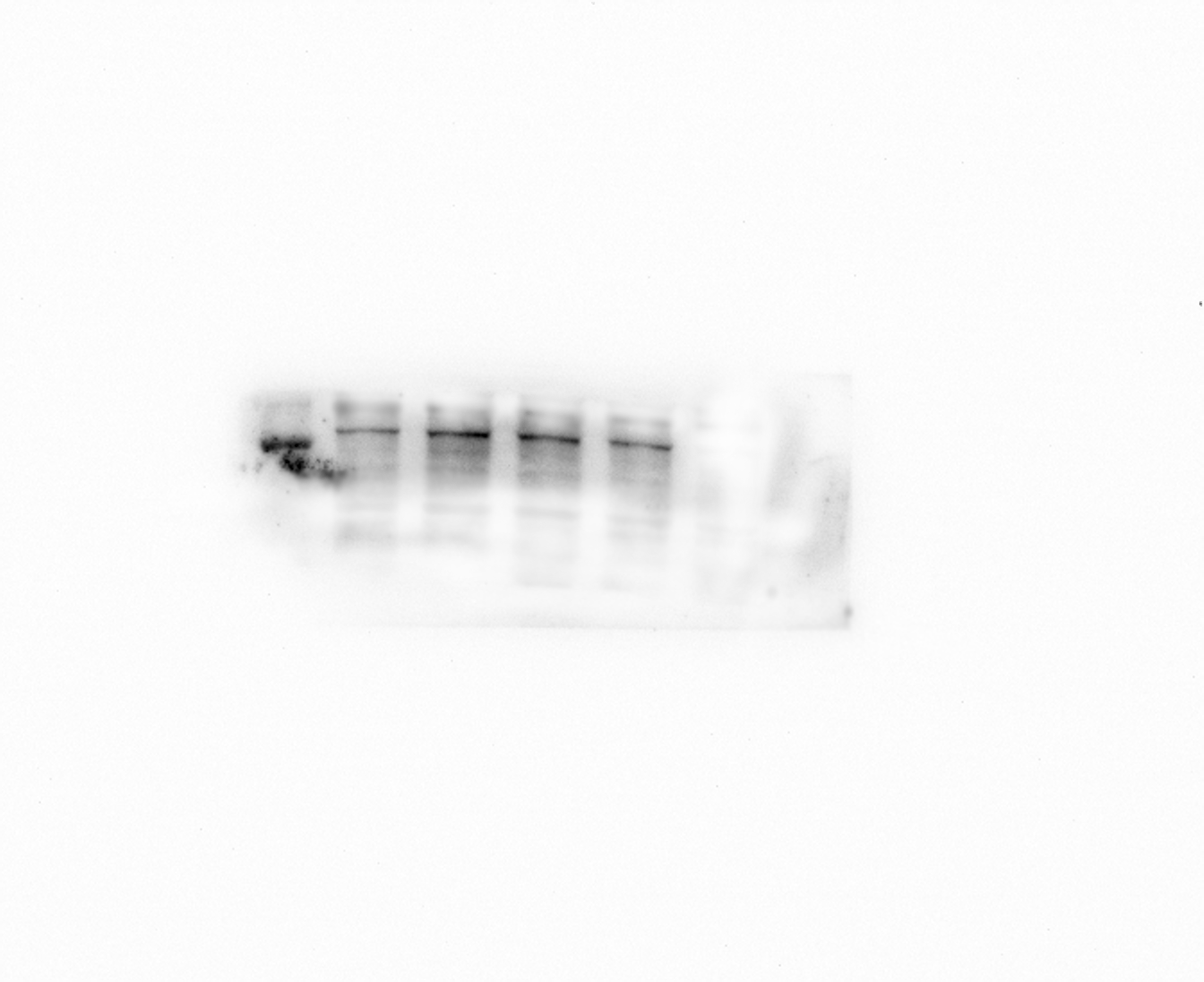

Supplement: Supplemental Information 5 [file peerj-08-8845-s005.tif]

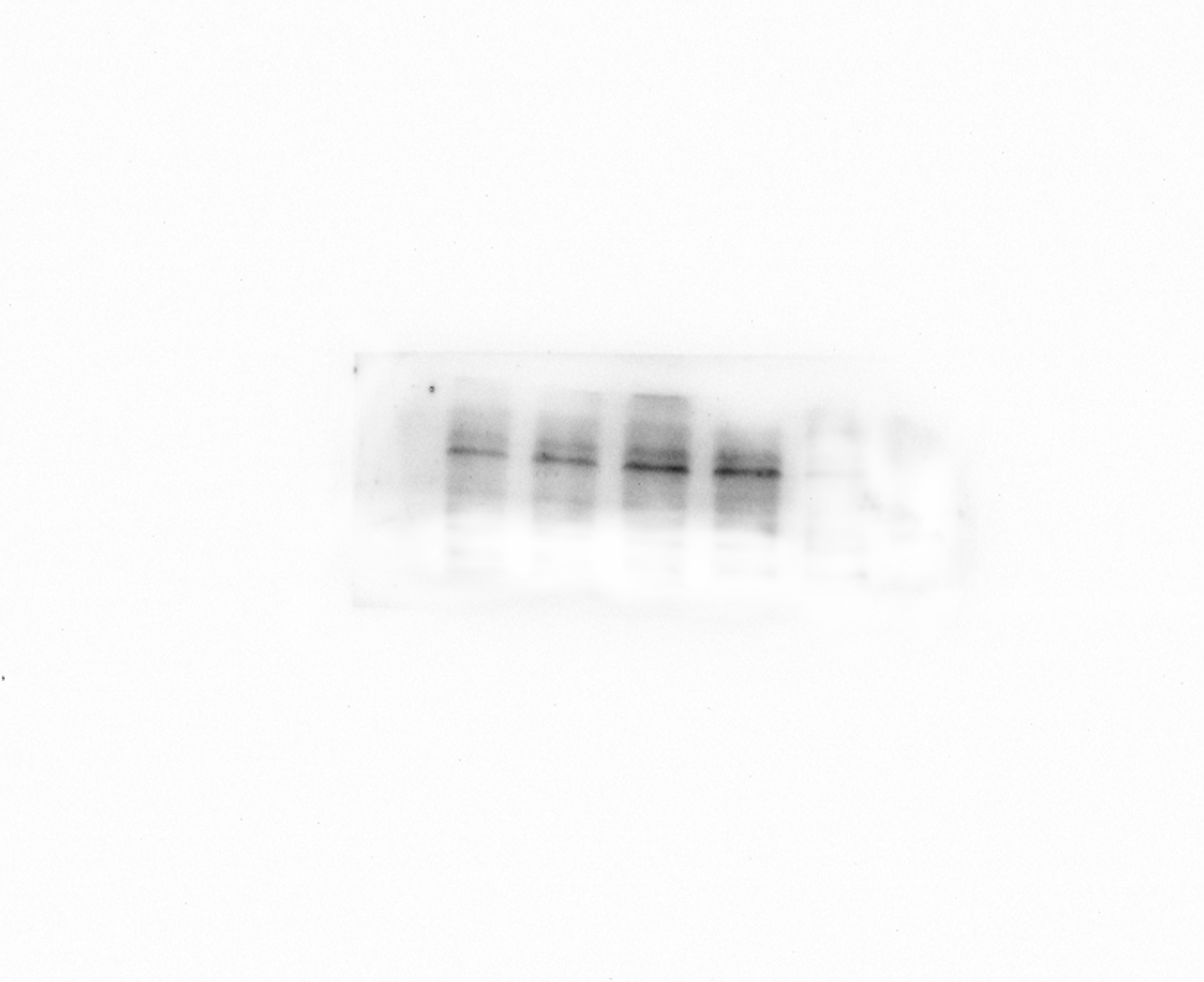

Supplement: Supplemental Information 6 [file peerj-08-8845-s006.tif]

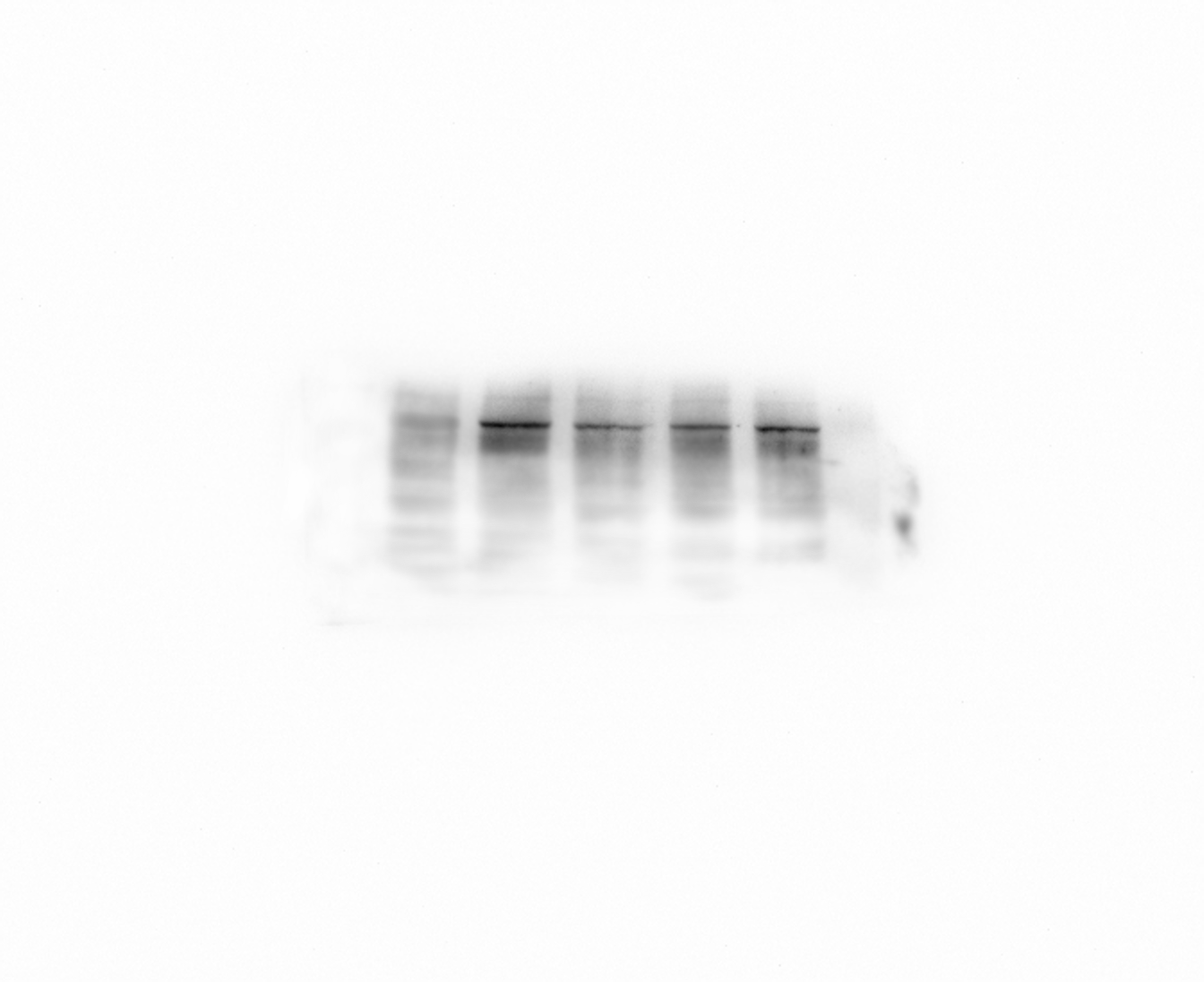

Supplement: Supplemental Information 7 [file peerj-08-8845-s007.tif]

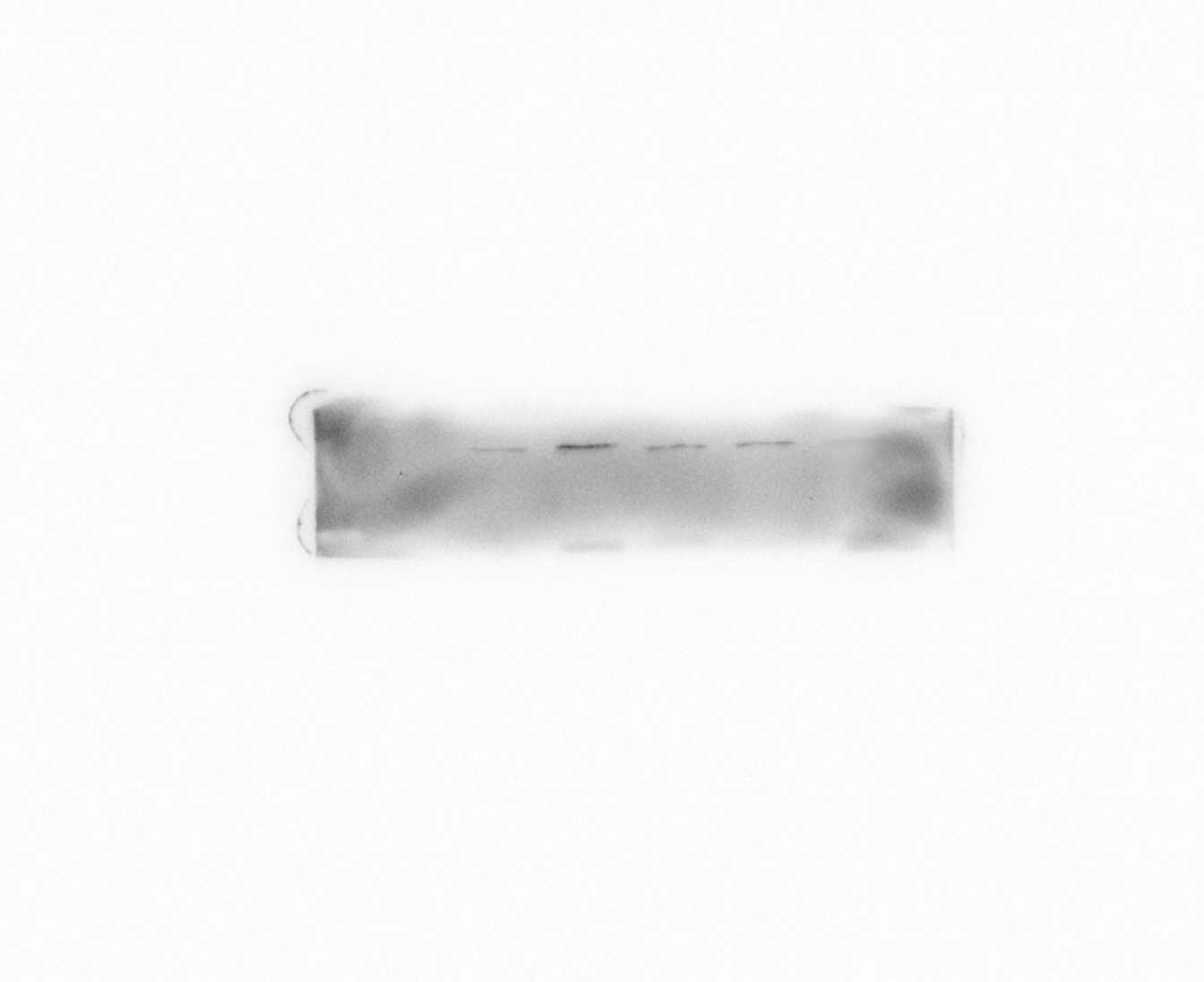

Supplement: Supplemental Information 8 [file peerj-08-8845-s008.tif]

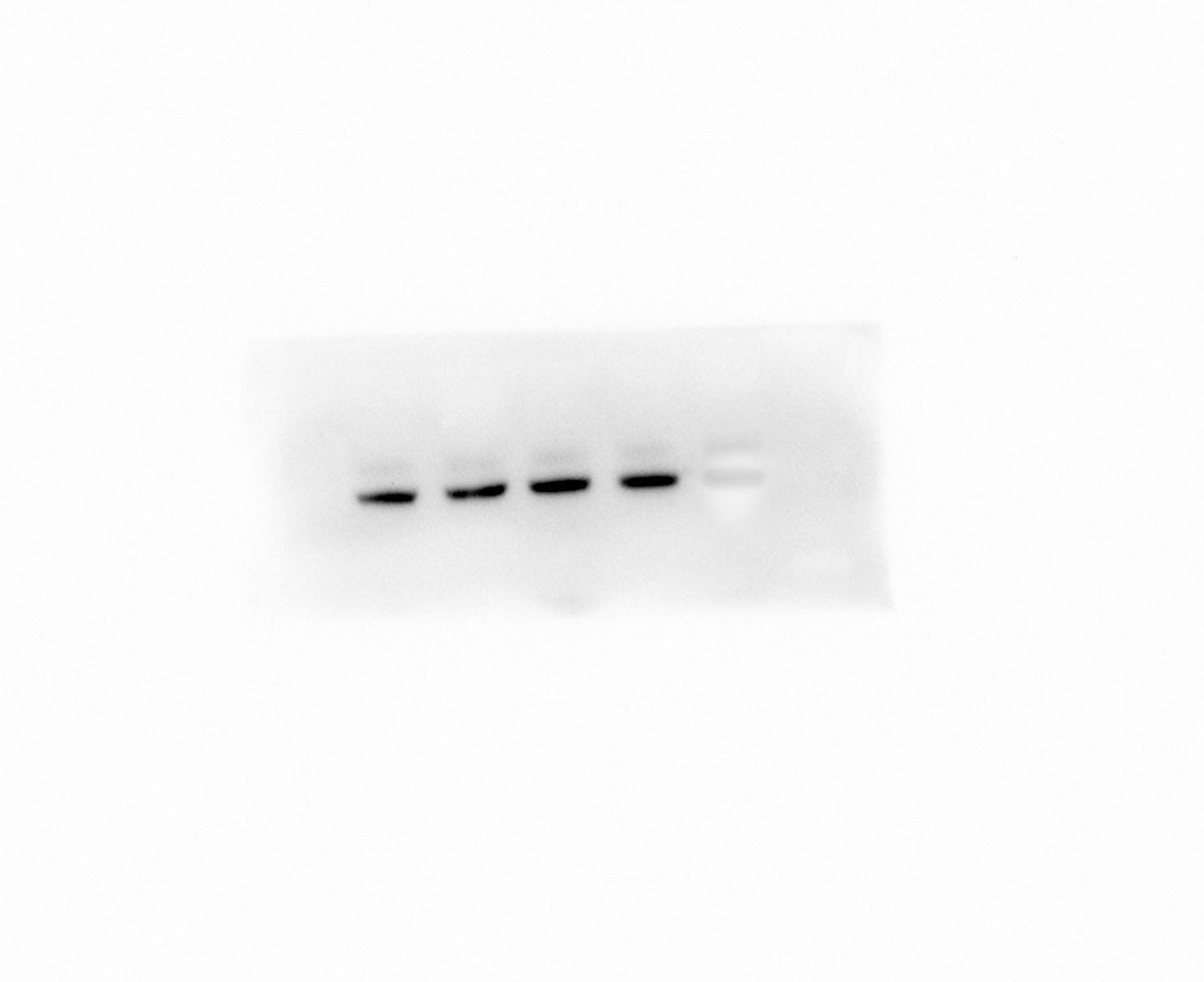

Supplement: Supplemental Information 9 [file peerj-08-8845-s009.tif]

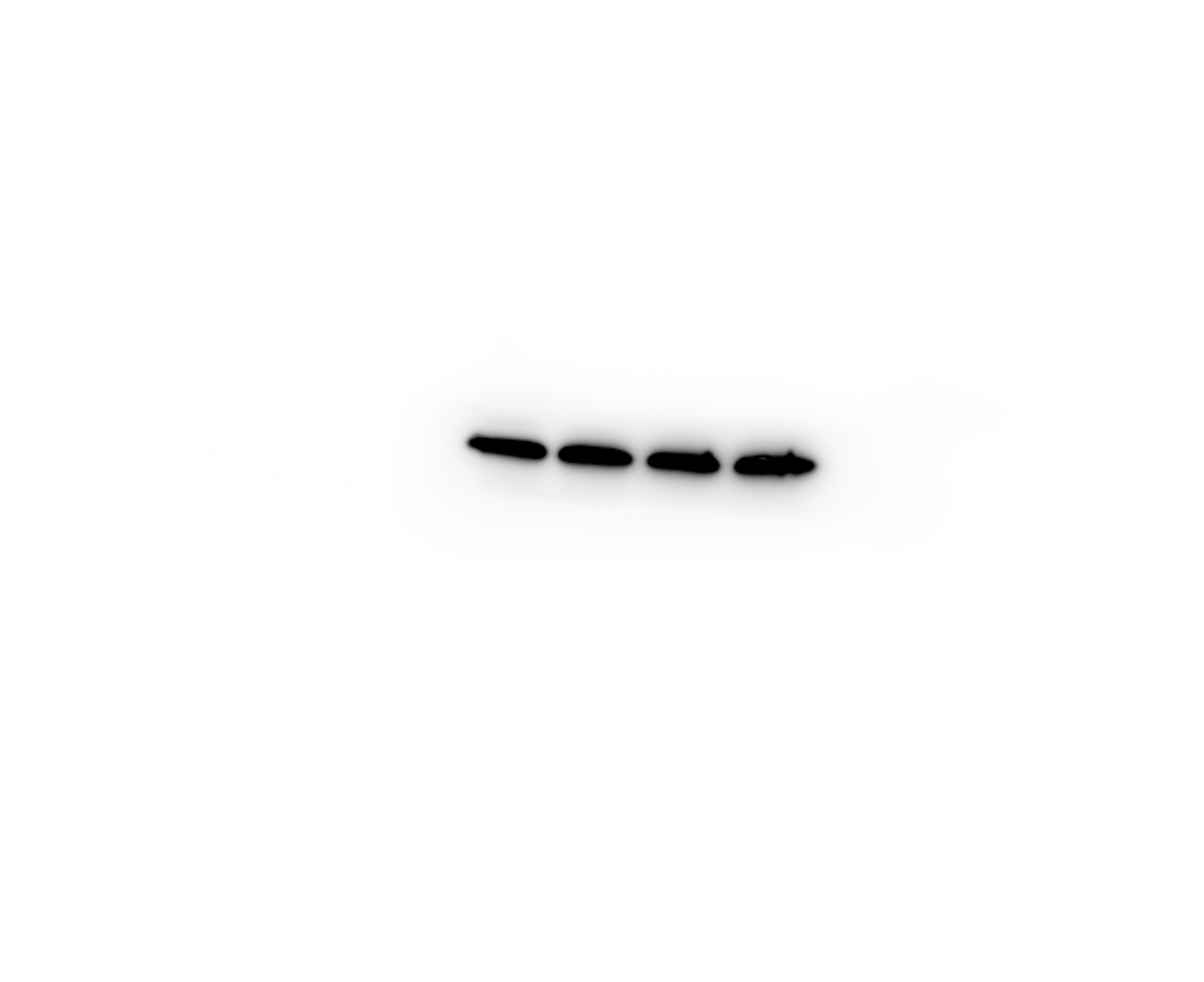

Supplement: Supplemental Information 10 [file peerj-08-8845-s010.tif]

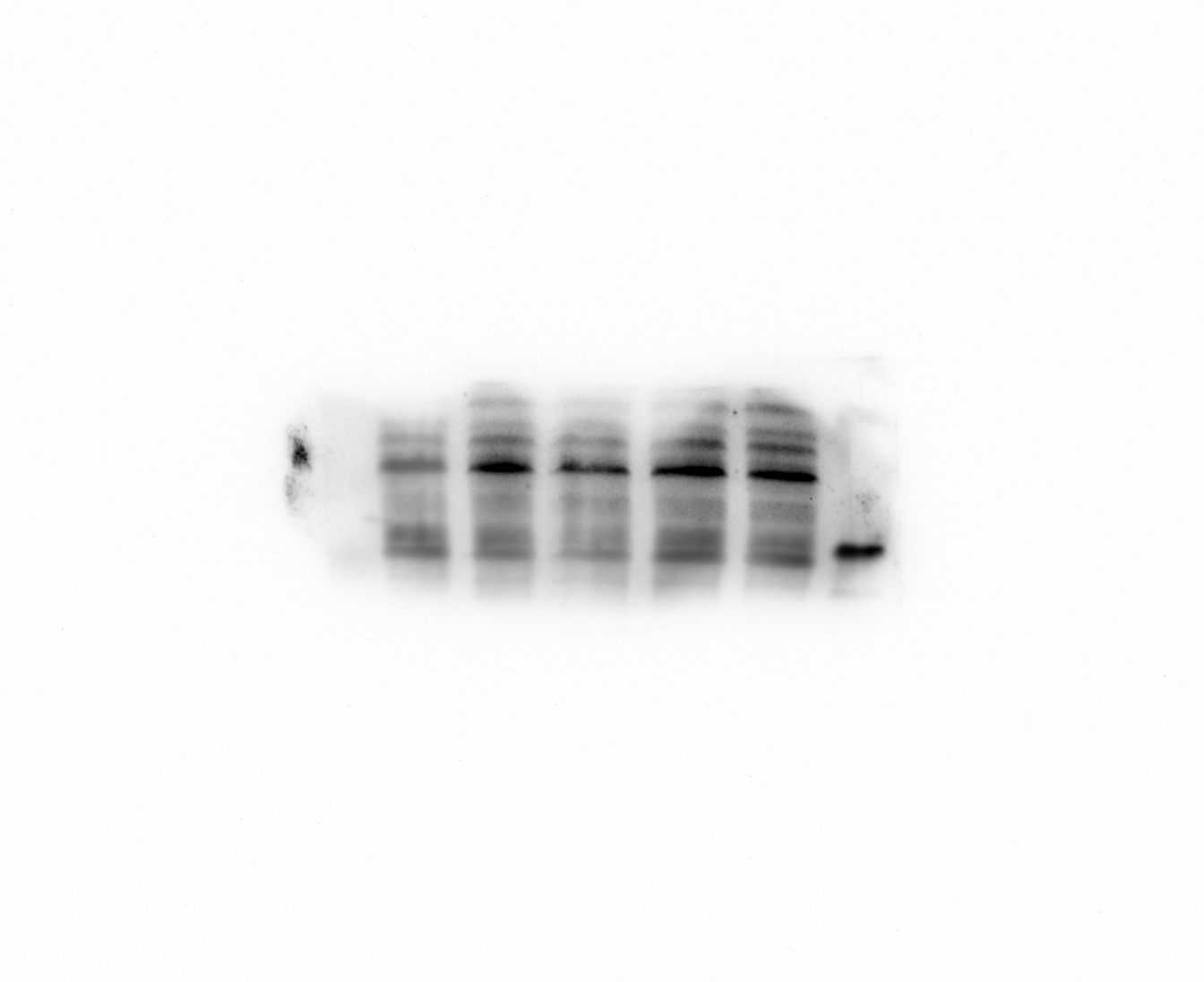

Supplement: Supplemental Information 11 [file peerj-08-8845-s011.tif]

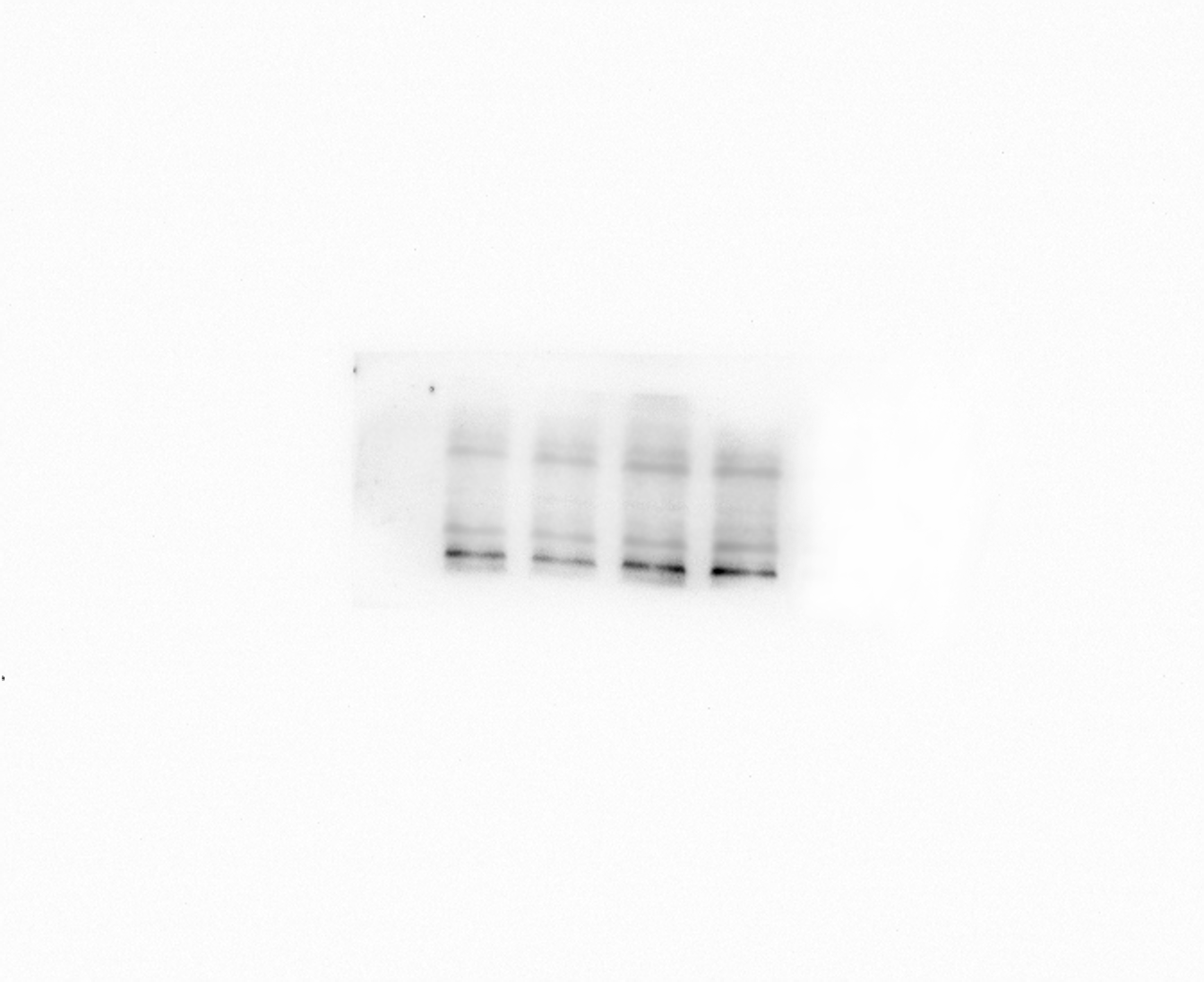

Supplement: Supplemental Information 12 [file peerj-08-8845-s012.tif]

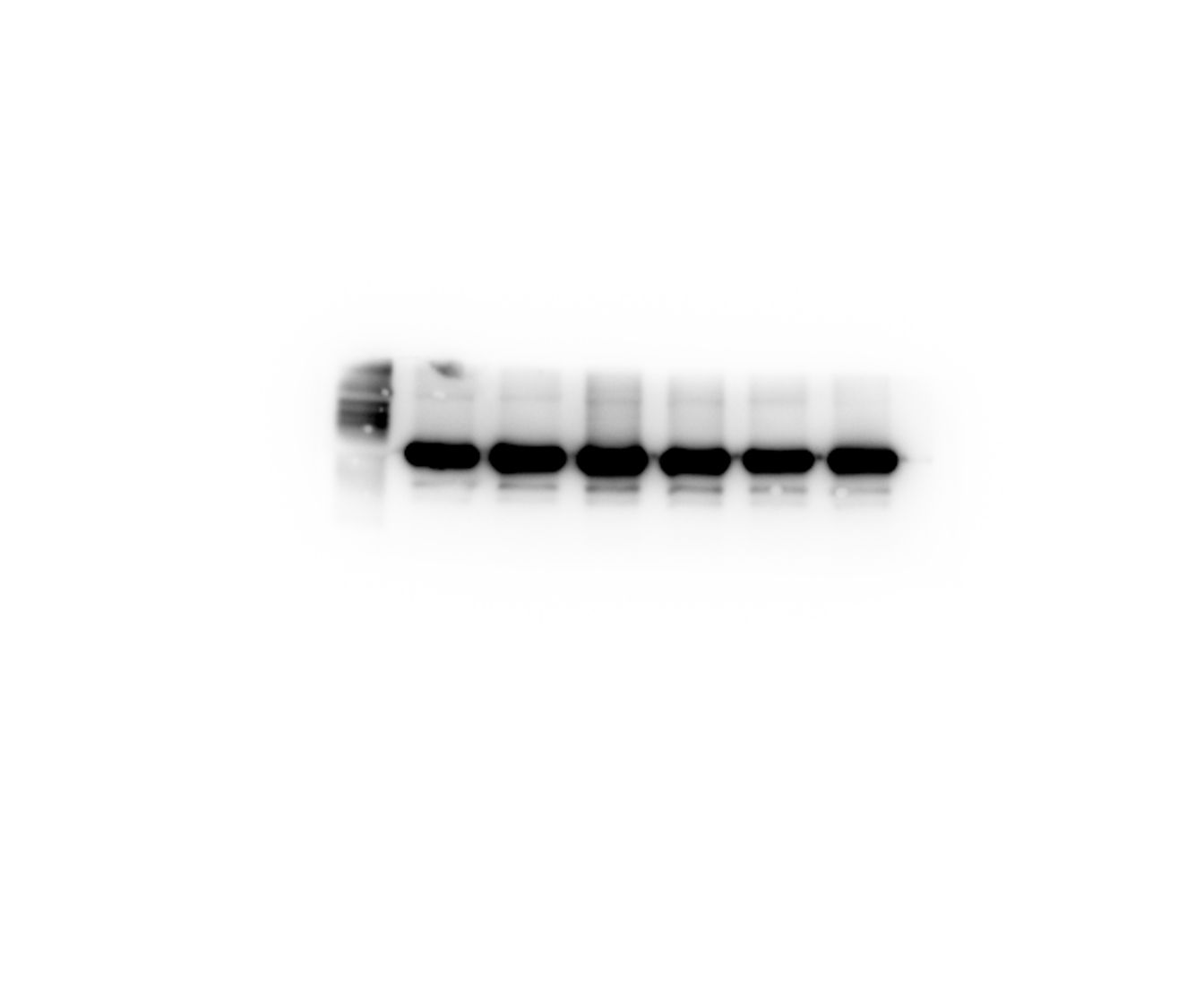

Supplement: Supplemental Information 13 — Only the first four bands were used in the experiment. [file peerj-08-8845-s013.tif]
